# The Impact of the COVID-19 Infodemic on Depression and Sleep Disorders: Focusing on Uncertainty Reduction Strategies and Level of Interpretation Theory

**DOI:** 10.2196/32552

**Published:** 2022-01-31

**Authors:** Soyoung Jung, Sooin Jung

**Affiliations:** 1 The School of Journalism and Communication Renmin University of China Beijing China; 2 Department of Kinesiology and Health Education College of Education The University of Texas at Austin Austin, TX United States

**Keywords:** COVID-19, social media, infodemic, construal level theory, uncertainty reduction strategy, depression, sleep disorder, preventive actions, affective reaction, infodemiology, misinformation, uncertainty, strategy, mental health, sleep, prevention, survey, usage, behavior

## Abstract

**Background:**

During the COVID-19 pandemic, information diffusion about the COVID-19 has attracted public attention through social media. The World Health Organization declared an infodemic of COVID-19 on February 15, 2020. Misinformation and disinformation, including overwhelming amounts of information about COVID-19 on social media, could promote adverse psychological effects.

**Objective:**

This study used the Psychological Distance and Level of Construal theory (CLT) to predict peoples’ negative psychological symptoms from social media usage. In this study, the CLT intended to show peoples’ psychological proximity to objects and events with respect to the COVID-19 pandemic. Furthermore, this study links the uncertainty reduction strategy (URS) and CLT for COVID-19–related preventive behaviors and affective reactions to assess their effects on mental health problems.

**Methods:**

A path model was tested (N=297) with data from a web-based survey to examine how social media usage behaviors are associated with URS and psychological distance with COVID-19 (based on the CLT), leading to preventive behaviors and affective reactions. Finally, the path model was used to examine how preventive behaviors and affective reactions are associated with mental health problems including anxiety and sleep disorder.

**Results:**

After measuring participants’ social media usage behavior, we found that an increase in general social media usage led to higher use of the URS and lower construal level on COVID-19. The URS is associated with preventive behaviors, but the CLT did not show any association with preventive behaviors; however, it increases affective reactions. Moreover, increased preventive behavior showed negative associations with symptoms of mental health problems; that is, depression and sleep disorder. However, the affective reaction tends to be positively associated with depression and sleep disorder. Owing to the infodemic of COVID-19, the psychological perception of the pandemic negatively influenced users’ mental health problems.

**Conclusions:**

Our results imply that the information from social media usage heightened concerns and had a lower construal level; this does not facilitate taking preventive actions but rather reinforces the negative emotional reaction and mental health problems. Thus, higher URS usage is desirable.

## Introduction

### Background

The COVID-19 pandemic is still ongoing worldwide after the World Health Organization (WHO) declared it a pandemic on March 11, 2020 [1]. Moreover, the second wave of the COVID-19 pandemic in European countries occurred in the summer of 2020 [2,3]. The WHO reported that most confirmed COVID-19 cases and deaths occurred in the United States [4]. COVID-19 introduced nonpharmaceutical interventions (NPIs) into people’s lives, which negatively impacted their everyday life, including activities such as working, studying, schooling, shopping, and dining [4-6]. A large majority of people’s everyday life changed to telecommuting (working remotely) and web-based learning. The NPI lifestyle pattern (ie, limited offline activities) have possibly impacted people’s psychological responses, including anxiety, depression, and sleep disorder [7].

For over 20 years in the history of pandemics—including the severe acute respiratory syndrome pandemic in 2002, A/H1N1 influenza pandemic in 2009-2010, and the Middle East Respiratory Syndrome pandemic in 2015, along with the current COVID-19 pandemic—health care workers’ psychological symptoms have been mostly examined [8,9]; however, relatively few studies focused on ordinary people’s psychological symptoms [10]. Compared to previous pandemic outbreaks, the COVID-19 pandemic has occurred uniquely in the age of social media. As a result of these changes and self-isolation, people’s social lives, especially their communication strategies, have experienced unprecedented changes. During self-isolation, people made videos of user-generated content (UGC) or pictures of their lives with the hashtags “#quarantine” and “#viewfromquarantine” [11]. Citizens in a state of confinement can experience psychological constraints and express fixation on the state of the disease and psychological disorders including sleep disorder and depression [8]. Social media usage led to the emergence of a novel situation called “infodemic” [12-17]. The impact of infodemics on social media users’ psychological perception—that is, construal level [18]—has been barely considered for the pandemic. Furthermore, the information contagion has a complex association with negative feelings; however, individual differences such as frequent social media usage and the uncertainty reduction strategy (URS) have not been considered.

How social media usage behavior and excess information regarding COVID-19 impact the users’ mental problems, including their URS (also known as their “information-seeking strategy”), still needs to be further examined. Since the onset of the pandemic, the following questions have been raised: (1) could social media usage increase concerns regarding COVID-19 and reduce the psychological distance between perceivers and COVID-19? (2) Among these associations, do individual differences such as URS impact further reactions such as preventive behavior, and do they help maintain mental health?

To answer these questions, we investigated how psychological symptoms are impacted by the URS and the perception of the Psychological Distance and Level of Construal theory (CLT) [19] during the COVID-19 pandemic [20]. We also examined how preventive behaviors and affective reactions are associated with the CLT and URS.

### CLT and Hypothetical Distance With Social Media Usage

The psychological distance that was examined with the CLT explains how people perceive an event or an object by their subjective feeling of distance. Trope and Liberman [21] defined the psychological distance as “the perception of when an event occurs, where it occurs, to whom it occurs, and whether it occurs.” Psychologically, a more distant form of an event or object is described as being more abstract and of a “higher construal” level. In contrast, a more proximal form of an object or event is shown to be more concrete and specific; that is, of a “lower construal” level [18,22]. This tendency can be applied to social perception [23], decision-making, and self-control [24]. In CLT research, known dimensions of the CLT are temporal, spatial, social, and hypothetical distance. Among them, hypothetical distance is mainly an applied dimension in the domain of consumer behavior research [13,25] and health communication in the context of mediated communication [26]. Hypothetical distance is based on *whether* the event could be happening or not, which implies “the likelihood of a target event happening” [27] or the probability level. The COVID-19 pandemic could be interpreted as a hypothetical dimension. As COVID-19 news and information is acquired from social media, frequent social media users perceive that they are closer to the event, and they feel that their community or residency is at a higher risk of COVID-19. Because the CLT describes how people experience psychological distance and how it affects attitudes, perception, and behaviors, the CLT has been considered a valuable framework for understanding a pandemic [20].

Altered news consumption patterns and UGC disseminations [28-31] have also been observed on social media during the COVID-19 pandemic. During emergency situations, social media could be a major channel of news consumption [28,30] and the primary source of news from neighbors and the community during the pandemic; for example, Nextdoor.com [32]. Three different types of altered patterns of information acquisition were observed: (1) mobile news adoption [29], (2) news consumption via social media, including Twitter [31], and (3) UGC contents from social media. The first case of mobile adoption allows people to consume the news incidentally, whenever and wherever they wish. The second pattern relates to news recommended by other users such as friends from social networking sites and a personalized recommendation system in social media platforms. In the third pattern, with emergent processes such as COVID-19 outbreaks, users have become news sources, creating their own news to elucidate their real-time situation.

Filtered and recommended news curated by computer recommendation systems is difficult to ignore. Such curated news accurately targets the users’ preferences on the basis of their social media usage behavior; that is, reading time, preferring topics, commenting, pressing “like” buttons, and following, including real-time topics [33] and user location [34]. Location information from location-based systems have facilitated studies on CLT and SNSs. For example, proximity effects (ie, geographic, social, and temporal proximity) on audiences’ expression of terrorism and the CLT approach have been studied during the Boston Marathon [35].

Furthermore, the impact of information consumption on social media about COVID-19 has been defined as an “infodemic” [13,17]—greater the amount of information regarding the pandemic acquired from social media, the more excessive perceived sensitivity among users. UGC helped information to be broadcasted in real time with regard to the COVID-19 outbreak—even faster than legacy media. This UGC showed devastating impacts of COVID-19; for example, how contagious COVID-19 could be, how quickly these infected people develop symptoms, and disease outcome including death. Thus, perceivers who see those UGC and circulated the news on social media about the pandemic may get frightened. Moreover, increased social media usage behavior may provide more chances of being exposed to more information and news about COVID-19. This may lead to greater concern among users with regard to the pandemic than among those who have not been exposed to social media.

Increased social media usage may lead to feelings of proximity to COVID-19, while social media users remain exposed to the updated information regarding the pandemic. Thus, their perception of the possibility of infection is higher; in other words, the hypothetical distance from COVID-19 is lesser than among those with lower social media usage.

Therefore, we propose the following research question (RQ) and hypothesis:

*RQ1:* To what extent does information acquisition on social media associate concerns regarding COVID-19 with CLT and URS?

*H1*: Higher information acquisition through social media usage is positively associated with the CLT on COVID-19.

### Preventive Behaviors and Emotional Reactions

Consuming more COVID-19 news through social media can significantly result in psychological proximity with the pandemic. Once individuals apply a lower construal level to the event, they use more concrete representations. They perceive the event as being more proximal to them, focusing on the *how* and evoking the negative emotion [20]. Conversely, the others have a higher construal level to the event, having more abstract representations and perceiving the event or object distantly from the self. A higher construal level is known to lead people to focus on the *why*. For example, if the pandemic occurred geographically distant from the perceivers (ie, other countries or continents) and had a time lag (ie, a couple months ago), one would think of social, political, and structural reasons, including the mechanism of contagion (ie, airborne aerosol-mediated transmission of SARS-CoV-2 [36]) and preventive methods (ie, wearing protective equipment including masks, gloves, and goggles and avoiding visiting public areas). In contrast, suppose the pandemic event occurred closer to the place recently. In that case, the perceivers are likely to focus on how to avoid a dangerous situation (eg, pay attention to other methods of contagion) and how to reserve everyday necessities (ie, food, water, toilet paper, and other essentials). Although people acquire information regarding how to prevent COVID-19, social media users could acquire deviant stories such as panic-buying at grocery stores or cases of rapid development of symptoms. Bowen [20] also explained how people interpret the pandemic and its impact on behaviors with an example of grocery shopping in a pandemic. Bowen [20] highlighted emotional evoking by each construal level; at higher construal levels, the abstracted and macro view evokes thankfulness during grocery shopping and thoughts on how the agricultural pipeline was maintained during the pandemic. However, the lower construal level approach generates anxiety among people on the issue of transmission from unknown sources or safety guidelines (ie, maintaining a 6-foot distance). When an individual becomes aware of the safety guidelines—for example, knowing that failing to maintain a 6-foot distance from others can increase the chance of infection—that person now not only knows how vital it is to maintain distance but also realizes the severity of COVID-19, which further increases anxiety. To extend this construal level to preventive behaviors, the lower construal level makes people focus on specific entities; in doing so, they tend to avoid the infection, but it is quite difficult for them to think about preventive behaviors associated with people with a higher construal level [20]. As Bowen [20] suggested, when people had a lower construal level, they reacted negatively and could not think of preventive behaviors.

To examine the application of CLT on the COVID-19 pandemic and the extent to which the lower construal level impact preventive behaviors and reinforce negative affective reactions, the following RQ and hypotheses are proposed.

*RQ2:* To what extent does construal level affect preventive behavioral actions (ie, wearing masks, using sanitizer, and avoiding attending public spaces)? Furthermore, to what extent does it reinforce affective reactions (ie, fear, difficulties, stress, and negative feelings)?

*H2A and H2B*: A lower construal level (shortened hypothetical distance) has (a) a negative association with preventive behaviors and (b) a positive association with emotional reactions.

However, though the participants were exposed to information regarding COVID-19, those with URSs could have preventive health behavioral information and may lower their concerns and negative psychopathological impacts such as those of depression, anxiety, and sleep disorder.

### Linking URS to Information-Seeking and Prevention Behaviors

The uncertainty reduction theory (URT) is used for information-seeking behaviors when uncertainties arise, utilizing many strategies to seek information about others [37] to reduce social anxiety caused by revealing users’ identity to those who are anonymous [38]. However, the extended URT has been applied to affection in mediated communication and the digital world [39] and in seeking health information [40]. When health-related information is not adequately acquired from the traditional medical professions, uncertainties arise, and then web-based information becomes the main source. Substituting the conventional information source, web-based information can be sought and shared by users to evaluate and verify [41]. Using the URS to information-seeking strategies, Berger [37] initially identified three types of knowledge-seeking techniques that an individual might use to minimize uncertainty: interactive, active, and passive knowledge-seeking techniques. First, interactive knowledge-seeking techniques are those in which the individual actively interacts directly with the target person and provides input. Second, active knowledge-seeking refers to whether an individual obtains information about a target from a second party that is more familiar with the target. Finally, passive knowledge-seeking techniques entail the person observing the target discreetly. Ramirez et al [42] explained extractive knowledge-seeking strategies, in which a person “draws upon a vast storehouse of written comments provided by targets” to acquire information and minimize confusion, using increasing information archived and retrievable on the internet [43]. Considering the URS in the current “infodemic,” the passive and extractive knowledge-seeking strategies may be applied. The passive and extractive knowledge-seeking strategy used here leads to examine how excessive social media usage may result in higher URS usage.

We argue that the URS in the communication domain is possible to link with the concern of COVID-19 outbreaks to obtain preventive knowledge. Furthermore, people with higher URS usage may have less emotional reactions, and having a URS leads to control mental health problems.

Therefore, we propose testing the following RQ and hypotheses:

*H3:* Increased information acquisition through social media usage is positively associated with URS usage.

*RQ3:* To what extent does the URS affect preventive behavioral actions (ie, wearing masks, using sanitizer, and avoid attending public space) and affective reactions (ie, fear, difficulties, stress, psychological burden, and negative feelings)?

*H4A and H4B*: The weighted URS shows a (a) positive association with preventive behaviors and (b) negative association with affective reactions.

### The Negative Psychological Symptoms: Depression and Sleep Disorder

The COVID-19 pandemic involves multiple factors such as an ever-increasing number of confirmed cases, depletion of protection equipment and daily necessity, feeling isolated, and lack of support. A combination of these multiple factors may instigate mental burden and the feeling of anxiety. However, most of those factors can be known through media coverage, including social media. Through social media usage, the acquired COVID-19 news and UGC result in shortening of the hypothetical distance with it, leading to negative emotional reactions such as stress, psychological burden, and anxiety. The feeling of stress and burden shows a higher association with psychological disorders—depression and sleep disorder.

During this critical COVID-19 situation, people are at risk of developing mental health symptoms. The multidimensional threatening situation can be known through widespread media coverage and social media. The sensational news from social media in users’ smartphones always delivers provocative and devastating news based on the personalized recommendation features of social media or recommendations by other users. However, altered information acquisition and consumption with social media and the mobile environment may result in a different psychological distance of construal level for the pandemic situation by the level of social media usage. Furthermore, individuals with a URS may be influenced to have more preventive information, leading to preventive behaviors such as wearing gloves and masks or avoiding attending public spaces. Those preventive behaviors may decrease the feeling of depression and sleep disorder. In sum, the overwhelmed information may contribute to these frequent social media users’ mental burden, while the URS reduces the mental health problem.

**Figure 1:**
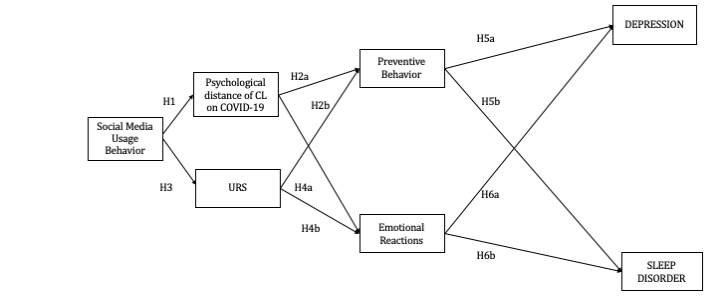
Proposed path model. CL: construal level; URS: uncertainty reduction strategy.

Therefore, we propose the following RQ and hypotheses that are indicated in the proposing path model of Figure 1:

*RQ4:* To what extent did the participants’ self-reported mental health symptoms—that is, depression and sleep disorder—were affected by COVID-19 and preventive behaviors?

*H5A and H5B:* Increased preventive behaviors of COVID-19 indicate negative associations with mental health symptoms: (a) depression and (b) sleep disorder.

*H6A and H6B:* Affective reactions on COVID-19 indicate positive associations with mental health symptoms: (a) depression and (b) sleep disorder.

## Methods

### Participants and Procedure

To measure the general populations’ perception of COVID-19 and their psychological response, we adopted a web-based cross-sectional survey through Amazon Mechanical Turk (AmTurk) to collect data. The Turk represents the general US population in terms of age and ethnicity more accurately than the college survey pool. Furthermore, the participants’ motivation and ability are better than those of the college sample [44-46].

Through the AmTurk advertisement, the participants could begin answering the web-based survey by clicking the participation agreement button on the web-based consent form. The survey questionnaire included questions on social media usage behavior, psychological distance on COVID-19, preventive behavior and emotional reactions, the Center for Epidemiology Scale for Depression (CES-D), and the Pittsburgh Sleep Quality Index (PSQI).

We used WarpPLS (version 7.0; ScriptWarp Systems) to not only examine the path model we proposed but also test the finesses of the hypothesized model.

### Measurements

The survey questionnaire included questions on social media usage behaviors, concern regarding COVID-19, a modified URS, a hypothetical distance of COVID-19, COVID-19 preventive behaviors, affective reactions, psychological symptoms, the CES-D, and the PSQI.

#### Social Media Usage Behavior

To measure social media usage behavior, we adopted suitable questions from questionnaires such as the Media Technology Usage and Attitude Scale [47], the Digital Natives Assessment Scale [48], and social media and digital natives. Example statements include “I send ‘links’ of contents to others using social media,” “I expect the social media that I visit regularly to be constantly updated,” and “I use the social media every day” (Cronbach α=.92). Answers were based on 7-point Likert scales (1=never, 7=always).

#### URS

The URS measurement [40] has been modified for this study; example statements include “I use the Internet and social media to find information regarding prevention for the COVID-19,” “I use the Internet and social media to find the symptoms of COVID-19 that were not discussed yet,” “I use the Internet and social media to seek alternative treatment or medical information regarding COVID-19 cure,” and “I use the Internet and social media to find the right information about the COVID-19” (Cronbach α=.87).

#### Psychological Distance of Construal Level on COVID-19

To measure the hypothetical distance of construal level on COVID-19, the question asked was “How is the COVID-19 situation in your residence area?” and the answers were provided on a 7-point Likert scale ranging 1=mild to 7=severe, 1=good to 7=bad, and 1=positive to 7=negative. For the question “Do you feel that your residential area is near to the impact of COVID-19?” the answers were based on a 7-point Likert scale ranging 1=near to 7-far (reversed coding was applied; Cronbach α=.87).

#### COVID-19 Preventive Behaviors

The preventive behaviors consist of the following statements: for example, “Worn a face mask,” “Washed/Sanitized hands,” “Worked or studied at home,” “Avoided in-person contact with high-risk people” (Cronbach α=.85). Their answers ranged 1=never to 7=always [49].

#### Emotional Reactions

To measure the participants’ affective reactions, the following questions were asked: “How often do you feel afraid of COVID-19?” “Do you feel that you are safe from the COVID-19?” “How often do you feel that you lack companionship?” “How often do you feel isolated from others?” “How often do you feel left out?” “How much has your sleep been interrupted or disturbed by concern about the outbreak?” and “How much difficulty do you have obtaining the food that you need because of the COVID-19 pandemic or social distancing rules?” (Cronbach α=.86), and the answers were based on 7-point Likert scales [49].

#### Depression (CES-D) and Sleep Quality (PSQI)

The CES-D was sued to measure depression symptoms, and the PSQI was used to measure sleep disorder symptoms. Detailed items and their reliability are reported in Tables 1 and 2.

### Statistical Power

Statistical power was estimated on the basis of the sample size. When a significance level of .05 (range .001 to –.50) was used, and the required power level was 0.80 (range 0-0.99), the minimum absolute significant path coefficient in the model was 0.15, the inverse square root methods required a minimum sample size of approximately 275 to run the path model, and the 297 participants of this study are above stipulated sample size threshold.

## Results

To examine how social media usage and individual differences are associated with the impact of psychological responses, a path model analysis using WarpPLS (version 7.0) [43-45] software was executed.

In total, 296 participants (106 male and 191 female) were included in the path model analyses. The ethnicity of participants included White (157/297, 52.9%), African American (45/297, 15.2%), Asian American (35/297, 12.1%), American Indian or Alaska Native (54/297, 18.2%), Native Hawaiian or Pacific Islander (2/297, 0.67%), and others (3/297, 1.01%). Their age distributions are reported in Table 1, and one of the participants refused to answer.

Measurements with items and their reliability are reported in Tables 2 and 3.

**Table 1:** Participant age distribution (N=296^a^).

Age (years)	Participants (N=296, 99.7%), n (%)
18-24	13 (4.6)
25-34	168 (56.6)
35-44	67 (22.6)
45-54	28 (9.4)
55-64	18 (6.1)
65-74	2 (0.7)
Total	296 (99.7)

^a^One (0.3%) missing participant refused to reveal his/her age.

**Table 2:** Items and reliability of mental health measurements.

Items			Cronbach α
**Center for Epidemiology Scale for Depression**			.93
	1. I was bothered by things that usually don’t bother me		
	2. I did not feel like eating; my appetite was poor		
	3. I felt that I could not shake off the blues even with help from my family or friends		
	4. I felt I was just as good as other people		
	5. I had trouble keeping my mind on what I was doing		
	6. I felt depressed.		
	7. I felt that everything I did was an effort		
	8. I felt hopeful about the future		
	9. I thought my life had been a failure		
	10. I felt fearful		
	11. My sleep was restless		
	12. I was happy		
	13. I talked less than usual		
	14. I felt lonely		
	15. People were unfriendly		
	16. I enjoyed life		
	17. I had crying spells		
	18. I felt sad		
	19. I felt that people dislike me		
	20. I could not get “going”		
**Pittsburgh Sleep Quality Index**			.87
	a. Cannot get to sleep within 30 minutes		
	b. Wake up in the middle of the night or early morning		
	c. Have to get up to use the bathroom		
	d. Cannot breathe comfortably		
	e. Cough or snore loudly		
	f. Feel too cold		
	g. Feel too hot		
	h. Have bad dreams		
	i. Have pain		

**Table 3:** Items and reliability of measurement.

Items				Cronbach α
**Social media usage**				.92
	Read and comment or write feedback (for example, on the opinion board, RT on Twitter or on the Facebook)			
	I send “links” to contents to others using social media			
	I habitually surf around information/contents			
	I surf around and click whatever get my attention			
	I do not particularly look for information/news and just “surf” habitually.			
	Watch TV shows, movies, etc. on a computer.			
	I feel it is important to be able to access the social media any time I want.			
	Technology will provide solutions to many of our problems.			
**Hypothetical distance: construal level on COVID-19**				.87
	**How is the COVID 19 situation in your residence area?**			
		Mild-severe		
		Good-bad		
		Positive-negative		
	Do you feel that your residence area is near to the impact of COVID-19? (Near-far^reversed^)			
	Do you feel that your neighbors are under the impact of COVID-19? (Impactful-not impactful^reversed^)			
	Do you feel that you are under the impact of COVID-19? (Near-far^reversed^)			
**Uncertainty reduction strategy**				.87
	I use internet and social media to find information regarding prevention for the COVID-19			
	I use internet and social media to find the symptoms of COVID-19 that were not discussed yet			
	I use internet and social media to seek alternative treatment or medical information regarding COVID-19 cure			
	I use internet and social media to find right information about the COVID-19			
**Preventive behaviors**				.85
	Worn a face mask			
	Washed/sanitized hands			
	Worked or studied at home			
	Cancelled/postponed work or school activities			
	Prayed			
	Avoided public places/crowds			
	Avoided in-person contact with high-risk people			
	Cancelled/postponed travel			
**Affective reactions**				.86
	How often do you feel that you lack companionship?			
	How often do you feel isolated from others?			
	How often do you feel left out?			
	How much has your sleep been interrupted or disturbed of concern about the outbreak?			
	How much difficulty do you have obtaining the food that you need because of the COVID-19 pandemic or social distancing rules?			

### Statistical Analysis

**Figure 2:**
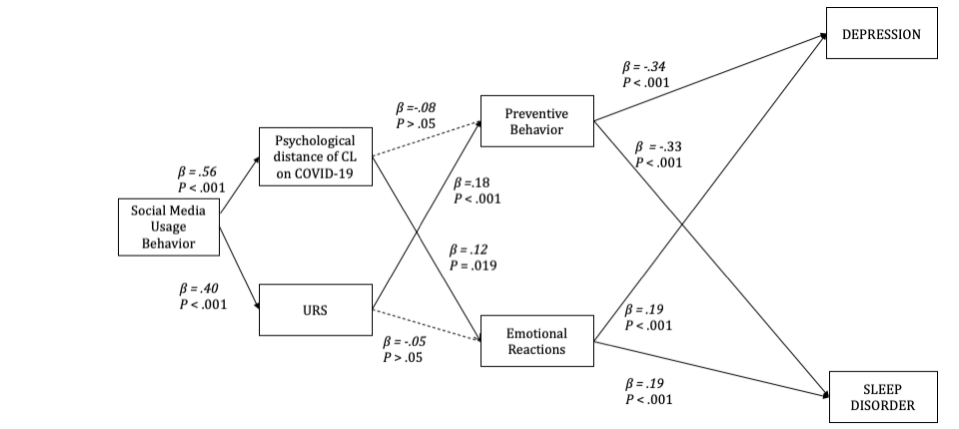
Analyzed path model. *P<.50, **P<.10. CL: construal level; URS: uncertainty reduction strategy.

Overall, path model analysis shows the goodness of fit average path coefficient (APC)=0.20, *P*<.001 and adjusted average R-squared (AARS)=0.08, *P*=.04, average variance inflation factor (AVIF)=1.014, which shows a good average block variance inflation factor (VIF). The proposed hypotheses testing and the results of the path model are available in Figure 2.

Social media usage increases URS usage (H3) and the hypothetical distance of construal level (H1). Both H1 and H3 are supported. The increased URS results in more preventive behaviors (H4A was supported), but it does not show an association with emotional reactions (H4B was not supported). In contrast, the psychological proximity on COVID-19 could not predict their preventive behaviors (H2A was not supported), but it predicted the emotional reactions (H2B was supported). The preventive behaviors positively associated with symptoms of mental health: depression and sleep disorder (H5A and H5B were supported). The participants’ greater affective reactions predicted the increased mental health symptoms: depression and sleep disorder (H6A and H6B were supported).

## Discussion

### Principal Findings

The principal objectives of this study were to examine the effects of social media usage on CLT and URS usage; while higher URS usage promoted the preventive behaviors, which reduced the self-reported mental health symptoms during the devastating COVID-19 outbreak situations, the CLT did not. Contrary to those with higher URS usage, those with low URS usage and emotional retraction showed increasing mental health symptoms, including depression and sleep disorders. Findings related to the underlying mechanism of mental health problems indicated several points regarding social media usage.

First, with increased social media usage, we predicted lower construal levels on COVID-19 (H1 was supported). By leveraging the CLT, we extended Lin et al’s [40] uncertainty reduction action from the health communication domain to the context of COVID-19 outbreaks with “infodemic.” Prior research on CLT used market research to predict the consumer behavior. However, we attempted to have a fresh perspective on CLT and extend its application area, such as the pandemic situation with preventive behaviors and evoking negative emotions. In doing so, hypothetical thoughts actually lead them to react emotionally; however, an increased probability of displaying preventive behavior was not observed. The lower construal level is known to be focused on the thought of “how” and specifics, but the social media usage and lower construal level could not consider aerosol transmission for COVID‐19, and it also fails to predict preventive behaviors; that is, wearing gloves, wearing masks, and washing hands. A possible explanation of this is that because the lower construal level narrows down the thoughts to the specifics, the participants focus on the specific incident and risks of the pandemic and not on transmission mechanism including behaviors to prevention. With higher construal, they have a broad perspective about the constructional perspective and its prevention. The preventive behaviors could be a target. In a target- or goal-oriented situation, higher construal results in an intervention being viewed in terms of a higher construal and thus increased goal commitment [50]. Therefore, the lower construal level did not show an association with preventive behaviors. Per our hypothesis (H2B), the participants indicated a lower construal level and showed negative emotional reactions owing to shortening of the hypothetical distance. The overwhelming information with their emotional response may deprive them of the chance to think about the situation objectively or cognitively. Thus, the emotional reaction leads to higher self-reported depression and sleep problems, resulting in a vicious circle. Both URS and CLT usage were predicted to help people maintain their mental health and take the necessary precautions. As a result, only URS usage has a positive impact, while the CLT increases anxiety regarding the COVID-19 pandemic.

Second, contrary to the hypothetical concept on COVID-19, increased URS usage with increased social media usage were positively related to preventive behaviors and were not associated with emotional reactions. The increased preventive behaviors indicated lower mental health symptoms, such as depression and sleep disorder, which implies a reduction in mental health problems with increased preventive behaviors. This finding implies that those with URS usage obtain proper information from social media, including the general internet, to maintain their healthy life rather than focusing on the negative emotional reactions—even in devastating situations. Higher social media usage and news recommendations, including popular UGC usage suggested in social media, are highly related to each other, which provide targeted information. Therefore, the users are hard to ignore. In this “infodemic” situation, the URS is more desirable to detect appropriate behaviors and maintain their mental symptoms.

### Limitations and Future Studies

Though our results are legitimate, their interpretation requires caution for the following reasons. First, the study sample showed a gender imbalance; 64.2% of the participants were female and 36.7% were male. To control the gender imbalance, the path analyses were controlled by the effects of gender imbalance. Second, though the participants displayed divergent racial and age distributions, the sample was collected only in the United States; hence, the generalizability of the results may be threatened. The sample and generalizability issues are common in academic studies. Future studies will expand our sampling context to a larger and diverse cross-section of the population; that is, other countries and nationalities. Furthermore, the sample bias on AmTurk was raised by Almaatouq et al [47]. In this study, though we used the AmTurk, convenience sampling may jeopardize the study’s reliability, this study attempts to reach a diverse group of social media users. AmTurk users have a greater proclivity for social media use. As a result, sampling bias is lesser than that of the overall school population. Moreover, the quality of data was relatively well-managed compared to that of other methods [48]. 

In addition, the results obtained in the context of COVID-19 are not directly similar to those obtained in the context of posttraumatic stress disorder (PTSD) or the effects of tragic events. However, in terms of experiencing sudden loss and isolation from others and financial problems, this is a novel situation akin to war and tragic, violent events that may cause PTSD [51-53]. Furthermore, the second effect of PTSD on the person’s family members may result from the person’s mental health problems [54]. As the self-isolation period gets prolonged, a higher rate of domestic violence has been reported [51,52,55]. Consequently, mental health issues may extend the secondary effects on their family, community, and society [56,57]. Therefore, future studies are required to investigate the effect of mental health problems due to the COVID-19 pandemic on family, community, and society.

### Conclusions

Considering the importance of preventing severe diseases and stopping contagion, psychological and mental issues are treated as secondary problems. However, our results imply that URS usage is associated with prevention behaviors and consequently, mental health problems tend to be managed. However, UGC on social media provides a chance to encourage people to live in a tragic situation by shortening the proximity with COVID-19, and it heightened the affective reactions reinforced mental health problems. The implications of this study are that while using social media, media literacy—that is, information-seeking behavior in the URS—is essential to maintain mental health during the self-isolation period and the pandemic situation to retain their psychological responses.

## Abbreviations

AmTurk: Amazon Mechanical Turk
CES-D: Center for Epidemiology Scale for Depression
CLT: Psychological Distance and Level of Construal theory
NPI: nonpharmaceutical intervention
PSQI: Pittsburgh Sleep Quality Index
PTSD: posttraumatic stress disorder
RQ: research question
UGC: user-generated content
URS: uncertainty reduction strategy
URT: uncertainty reduction theory
WHO: World Health Organization
